# Folliculin regulates cell–cell adhesion, AMPK, and mTORC1 in a cell‐type‐specific manner in lung‐derived cells

**DOI:** 10.14814/phy2.12107

**Published:** 2014-08-13

**Authors:** Damir Khabibullin, Douglas A. Medvetz, Miguel Pinilla, Venkatesh Hariharan, Chenggang Li, Anja Hergrueter, Maria Laucho Contreras, Erik Zhang, Andrey Parkhitko, Jane J. Yu, Caroline A. Owen, Hayden Huang, Rebecca M. Baron, Elizabeth P. Henske

**Affiliations:** 1 Division of Pulmonary and Critical Care Medicine, Brigham and Women's Hospital, Boston, Massachusetts; 2 Harvard Medical School, Boston, Massachusetts; 3 Department of Biomedical Engineering, Columbia University, New York City, New York; 4 The Lovelace Respiratory Research Institute, Albuquerque, New Mexico

**Keywords:** AMPK, BHD, Cell–cell adhesion, folliculin, mTOR, pneumothorax

## Abstract

Germline loss‐of‐function BHD mutations cause cystic lung disease and hereditary pneumothorax, yet little is known about the impact of *BHD* mutations in the lung. Folliculin (FLCN), the product of the Birt–Hogg–Dube (BHD) gene, has been linked to altered cell–cell adhesion and to the AMPK and mTORC1 signaling pathways. We found that downregulation of FLCN in human bronchial epithelial (HBE) cells decreased the phosphorylation of ACC, a marker of AMPK activation, while downregulation of FLCN in small airway epithelial (SAEC) cells increased the activity of phospho‐S6, a marker of mTORC1 activation, highlighting the cell type–dependent functions of FLCN. Cell–cell adhesion forces were significantly increased in FLCN‐deficient HBE cells, consistent with prior findings in FLCN‐deficient human kidney‐derived cells. To determine how these altered cell–cell adhesion forces impact the lung, we exposed mice with heterozygous inactivation of *Bhd* (similarly to humans with germline inactivation of one BHD allele) to mechanical ventilation at high tidal volumes. *Bhd*^+/−^ mice exhibited a trend (*P* = 0.08) toward increased elastance after 6 h of ventilation at 24 cc/kg. Our results indicate that FLCN regulates the AMPK and mTORC1 pathways and cell–cell adhesion in a cell type–dependent manner. FLCN deficiency may impact the physiologic response to inflation‐induced mechanical stress, but further investigation is required. We hypothesize that FLCN‐dependent effects on signaling and cellular adhesion contribute to the pathogenesis of cystic lung disease in BHD patients.

## Abstract (precis)

We found that downregulation of FLCN in human bronchial epithelial (HBE) cells decreased the phosphorylation of ACC, a marker of AMPK activation, while downregulation of FLCN in small airway epithelial (SAEC) cells increased the activity of phospho‐S6, a marker of mTORC1 activation, highlighting the cell type–dependent functions of FLCN. Cell–cell adhesion forces were significantly increased in FLCN‐deficient HBE cells, consistent with prior findings in FLCN‐deficient human kidney‐derived cells. To determine how these altered cell–cell adhesion forces impact the lung, we exposed mice with heterozygous inactivation of Bhd (similarly to humans with germline inactivation of one BHD allele) to mechanical ventilation at high tidal volumes. Bhd+/− mice exhibited a trend (*P* = 0.08) toward increased elastance after 6 h of ventilation at 24 cc/kg.

## Introduction

Birt–Hogg–Dube (BHD) syndrome is an autosomal dominant tumor suppressor gene syndrome associated with cystic lung disease, spontaneous pneumothorax (lung collapse) which can be life‐threatening, hamartomatous skin tumors (fibrofolliculomas), and kidney cancer, which can include chromophobe and chromophobe‐oncocytic hybrid tumors (Zbar et al. 2002; Toro et al. 2008; Menko et al. 2009). It is estimated that 35% of BHD patients have a family history of pneumothorax (Gupta et al. 2013). Germline *BHD* mutations are the most common cause of hereditary pneumothorax even in patients without evidence of renal or skin lesions (Painter et al. 2005; Frohlich et al. 2008; Ren et al. 2008; Sundaram et al. 2009). Cystic lung disease in BHD patients has been observed in utero (Sundaram et al. 2009) and pneumothorax as young as age 16 (Furuya and Nakatani 2013). Biopsy specimens from BHD patients have revealed that the pulmonary cysts are closely associated with the interlobular septa or visceral pleura and are lined by a layer of alveolar epithelium, distinguishing them from other types of bullous changes (Koga et al. 2009). Increased angiogenesis surrounding the epithelial lined cystic structure with increased expression of hypoxia‐inducible factor 1 (Nishii et al. 2013) suggest that cystic disease in BHD may result in part from an angiogenic stimulus. Interestingly, recent analysis of the largest cohort of BHD lung specimens to date suggests that cyst formation may be triggered by mechanical forces generated during respiration, leading to disruption of alveolar homeostasis (Kumasaka et al. 2014).

Nearly all *BHD* mutations lead to premature truncation of FLCN, a 64 kDa protein with no obvious homology to other human proteins (Nickerson et al. 2002; Graham et al. 2005; Painter et al. 2005; Schmidt et al. 2005; Vocke et al. 2005). Recent structural modeling of FLCN has revealed a domain with guanine exchange factor activity toward the small G protein Rab35 (Nookala et al. 2012). Loss of FLCN disrupts the polarized growth of kidney‐derived (Luijten et al. 2013) and colon cancer‐derived cells (Medvetz et al. 2012). Kidney cells and fibroblasts lacking FLCN have an increase in S phase cells (Laviolette et al. 2013), and FLCN‐deficient HeLa cells have increased cyclin D1 levels (Kawai et al. 2013), indicating that enhanced cellular proliferation is a consequence of mutational inactivation of BHD at least in some cell types. FLCN‐deficient muscle and kidney cells have striking increases in mitochondrial number (Hasumi et al. 2012). FLCN has been shown to regulate multiple cellular signaling networks. FLCN regulates the activity of the AMPK and mTOR pathways (Okimoto et al. 2004; Baba et al. 2006, 2008; van Slegten‐horst et al. 2007; Chen et al. 2008; Hartman et al. 2009; Hong et al. 2010; Hudon et al. 2010; Cash et al. 2011) in kidney‐derived cells, fibroblasts, and muscle‐derived cells, with the majority of studies showing FLCN‐deficient cells have decreased levels of mTORC1 activity (Hartman et al. 2009; Hasumi et al. 2012; Bastola et al. 2013). However, some FLCN‐deficient cells, both in vitro and in vivo, show elevated levels of mTORC1 activity, leading to speculation that FLCN's effects on cellular signaling are highly context dependent. Most recently, FLCN has been found to localize to lysosomes in an amino acid–dependent fashion and to regulate mTORC1 activity on the lysosomal membrane (Petit et al. 2013; Tsun et al. 2013).

FLCN has broad effects on gene transcription in kidney‐derived cells and fibroblasts, including TGF‐beta components and targets (Hong et al. 2010; Cash et al. 2011). There are two mechanisms through which FLCN may regulate gene transcription. First, FLCN‐deficient renal cells have increased levels of ribosomal RNA synthesis through a physical interaction between FLCN and RPT4 (Gaur et al. 2013). Second, BHD was recently identified in a large‐scale siRNA screen as an essential gene for embryonic stem cell commitment through regulation of the nuclear/cytoplasmic localization of the transcription factor Tfe3, with FLCN‐deficient cells showing increased nuclear Tfe3 (Betschinger et al. 2013).

We and others have recently discovered a direct physical interaction between FLCN and Plakophilin 4 (PKP4, also called p0071) (Medvetz et al. 2012; Nahorski et al. 2012). PKP4 is a member of the armadillo repeat containing protein family that also includes beta‐catenin and p120 catenin. PKP4 binds E‐cadherin at adherens junctions and regulates the activity of RhoA in the cytoplasm. Consistent with its interaction with PKP4, FLCN regulates Rho signaling in thyroid (Nahorski et al. 2012) and kidney‐derived cells (Medvetz et al. 2012). In addition, we found that FLCN‐deficient HEK293 cells and UOK‐257 cells, which are derived from a BHD patient's renal carcinoma, display a striking increase in cell‐–cell adhesion relative to control FLCN‐expressing cells. Cell–cell adhesion forces are also increased in PKP4‐deficient cells, consistent with a model in which FLCN is a negative regulator of cell–cell adhesion via its interaction with PKP4. Recently, FLCN‐deficient mouse alveolar epithelial cells were found to have low levels of E‐cadherin (Goncharova et al. 2014), suggesting that cell–cell junctions may be important to the lung phenotypes of BHD.

Here, we investigated for the first time the impact of FLCN deficiency on cellular signaling and cell–cell adhesion forces of human lung‐derived cells. We also investigated the in vivo pulmonary physiology of *Bhd*^+/−^ mice, which have inactivation of one allele of the *Bhd* gene. These mice provide a model for human BHD patients who have germline inactivating mutations of one allele of the *BHD* gene. We found that FLCN deficiency in lung‐derived cells induces some phenotypes that are shared with FLCN‐deficient kidney cells, but with clear cell‐type‐specificity between HBE and SAEC cells. The cell adhesion phenotype of FLCN‐deficient HBE cells is particularly interesting because cell–cell junctions are essential to the intrinsic architecture and stability of epithelial structures.

## Materials and Methods

### Cell culture and transfections

HBE and SAEC cells were obtained from Lonza and maintained in SABM media according to the manufacturer's protocol (Hopkinton, MA). HeLa and HEK293 cells were purchased from ATCC (Manassas, VA) and were maintained in DMEM‐high glucose media supplemented with 10% FBS and 1% pen/strep. FLCN downregulation in lung‐derived HBE and SAEC was achieved using shRNA lentiviral particles (Sigma, St. Louis, MO). Cells stably expressing shRNA were selected using 2 ug/ml puromycin. Proliferation was measured using crystal violet staining.

### Immunoblotting

Cells were lysed on ice in 1× RIPA buffer and centrifuged at 10,000 g for 15 min. The supernatants were boiled with LDS sample buffer and proteins separated using 4–12% NuPage gels (Life Technologies, Grand Island, NY). Antibodies used in the current study: CREB, FLCN, phospho‐ACC (S79), pS6 (Ser 235/Ser236), phospho‐MAPK p44/42 (T202/Y204), cofilin, and phospho‐cofilin (Ser 3) antibodies (Cell Signaling, Danvers, MA); p62, actin and p120 catenin (Sigma); PKP4 (Progen Biotechnik, Heidelberg, Germany); GAPDH (Abcam, Cambridge, MA). Chemiluminescent signals were captured using a Syngene G‐BOX iChemi XT imager and quantified with Syngene GeneTools software (Syngene, Cambridge, U.K.).

### Cytoplasmic/nuclear fractionation

Cytoplasmic and nuclear fractions were isolated using a CelLytic NuCLEAR extraction kit (Sigma). Antibodies against GAPDH and CREB were used as controls for cytoplasmic and nuclear fractions, respectively.

### Cellular aggregation assay

The cell aggregation assay to measure cell–cell adhesion was adapted from previously described methods (Medvetz et al. 2012). Aggregate size was quantified using ImageJ and the Integrated Morphometry Analysis feature in Metamorph (Sunnyvale, CA).

### Real‐time quantitative PCR (qRT‐PCR)

Total RNA was extracted using the RNeasy mini RNA Isolation Kit (Qiagen, Valencia, CA). Commercial TaqMan probes and One‐step RT‐PCR mix (Applied Biosystems, Life Technologies) were used. Assays were performed on the Applied Biosystems Step One Plus instrument.

### Analysis of airspace enlargement in mice

All animal experiments were carried out under guidelines approved by Boston Children's Hospital Institutional Animal Care and Use Committee (IACUC). Bhd^+/−^ mice in the A/J background were generated by backcrossing Bhd^+/−^ C57BL6 mice into A/J background for six generations. For analysis of distal airspace size, mice were euthanized, the lungs were inflated to 25 cm H_2_O pressure, removed, and fixed in 10% saline‐buffered formalin. Midsagittal lung sections (8 *µ*m) were stained with Gill's stain, and 10–15 images of the lung sections (×200 magnification) were captured in a randomized and blinded fashion using a Leica microscope and QWin Leica Software. Scion Image software (Scion Corp., Frederick, MD) was used to measure alveolar chord length (in microns) and mean alveolar area (in microns^2^) (Moghadaszadeh et al. 2013).

### Lung physiology

8–10 week old male mice (C57BL6 background) were anesthetized with pentobarbital 60 *μ*g/g intraperitoneally, tracheotomized, and subjected to ventilator‐induced lung injury on the mouse Flexivent ventilator (Scireq, Tempe, AZ) using 24 mL/kg of tidal volume, with a respiratory rate of 150 breaths/min, and PEEP 2.5 on room air for 6 h. The mice were monitored continuously to maintain a surgical plane of anesthesia, and hourly measurements of lung physiology were performed after a recruitment maneuver. Following completion of mechanical ventilation, BAL fluid was collected by instilling and recovering 1 mL of saline three times into the tracheostomy tube. Recovered BAL volume was measured, and results were normalized to account for the percentage of recovered volume. BAL supernatant was recovered after centrifugation at 235 *g* for 5 min. Cell counts were performed using a hemocytometer. A cytospin was made using 200 *µ*L of the resuspended cells, stained with DiffQuik (Fisher Scientific, Pittsburgh, PA), then assessed for percentages of neutrophils, macrophages, lymphocytes, eosinophils, and basophils by manually counting three randomly selected fields. BAL IL‐6 levels were measured using an IL‐6 ELISA kit (eBioscience, San Diego, CA), and BAL protein levels were measured using the Bradford dye‐binding method (Bio‐Rad, Hercules, CA). Analysis of distal airspace size in mice after ventilation was performed as described above.

## Results

### FLCN localizes primarily to the cytoplasm and does not affect proliferation in vitro

**Figure 1.**
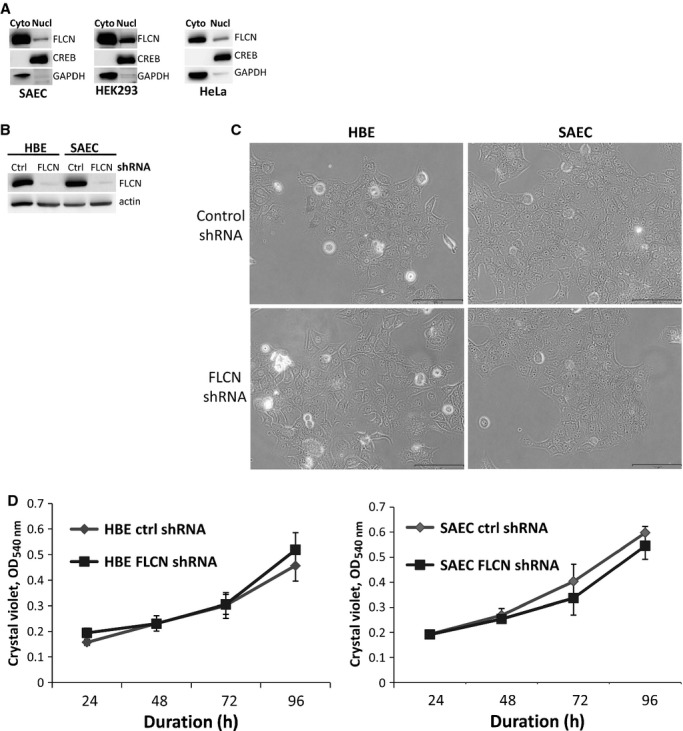
FLCN primarily localizes to the cytoplasm and does not regulate proliferation of lung‐derived cells. (A) Nuclear and cytoplasmic fractions were prepared from SAEC, HEK293, and HeLa cells. FLCN localized primarily to the cytoplasm of all three cell types. CREB was used as a control for the nuclear fraction and GAPDH as a control for the cytoplasmic fraction. Equal amounts of protein in each fraction were loaded. Each panel represents a separate gel. (B) FLCN was downregulated in HBE and SAEC cells using shRNA. Downregulation of FLCN was confirmed by immunoblot. (C) No obvious change in cellular morphology was observed. (D) No difference in cell doubling was observed over 96 h between HBE and SAEC cells expressing control shRNA versus FLCN shRNA.

FLCN has been reported to localize to both the cytoplasm and the nucleus, with some cell types including kidney showing predominantly nuclear localization (Laviolette et al. 2013) while embryonic stem cells show almost exclusively cytoplasmic localization (Betschinger et al. 2013). We used cellular fractionation to evaluate the nuclear versus cytoplasmic localization of FLCN in SAEC cells compared with HEK293 cells and HeLa cells. FLCN was predominately cytoplasmic in all three cell types (Fig. 1A).

In some patients, the pulmonary cystic lesions in BHD are epithelial lined (Koga et al. 2009; Furuya et al. 2012), suggesting that the cysts may result from hyperproliferation of cells with FLCN mutations. To determine whether FLCN regulates the proliferation of lung‐derived cells, we used shRNA to stably downregulate FLCN in human bronchial epithelial (HBE) and small airway epithelial cells (SAEC). Downregulation of FLCN was confirmed by western immunoblot (Fig. 1B). No obvious changes in cellular morphology were observed by phase contrast microscopy, with both control shRNA and FLCN shRNA expressing cells exhibiting a typical epithelial cobblestone appearance (Fig. 1C). Additionally, no difference in cell doubling was detected over 96 h using crystal violet staining (Fig. 1D).

### Impact of FLCN downregulation on cellular signaling in HBE and SAEC cells

**Figure 2.**
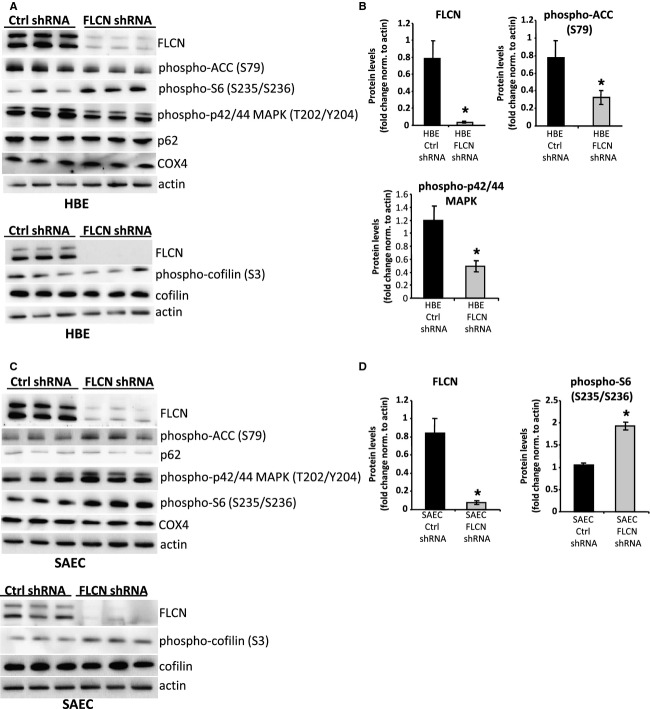
Impact of FLCN downregulation on cellular signaling in lung‐derived cells. (A, B) HBE cells with FLCN downregulation or control shRNA (three biologic replicates per shRNA cell line) were immunoblotted for proteins and phospho‐proteins in pathways known to be regulated by FLCN in other cellular lineages, including AMPK (phospho‐ACC), mTORC1 (phospho‐S6), autophagy (p62/sequestosome1), mitochondrial biogenesis (Cox4), and Rho Kinase (phospho‐cofilin). *P < 0.05 (C, D) SAEC cells with FLCN downregulation or control shRNA (three biologic replicates per shRNA cell line) were immunoblotted for the same panel of proteins and phospho‐proteins as in Figure 2A. *P < 0.05

FLCN has been shown to regulate multiple cellular signaling pathways in kidney cancer‐derived cells, fibroblasts, HEK293 cells, and thyroid cancer‐derived cells, including signaling networks involving mTOR, AMPK, Rho Kinase, and mitochondrial biogenesis (Baba et al. 2006, 2008; van Slegten‐horst et al. 2007; Hartman et al. 2009; Hasumi et al. 2009; Hudon et al. 2010; Medvetz et al. 2012; Nahorski et al. 2012; Bastola et al. 2013). In HBE cells with FLCN downregulation, we observed an approximately twofold decrease in the phosphorylation of ACC, a target of AMPK, consistent with recent observations in mouse alveolar epithelial cells (Goncharova et al. 2014). No significant change in the phosphorylation of ribosomal protein S6 (a marker of mTORC1 activity), p62/sequestosome 1 (an autophagy indicator and also a critical regulator of mTORC1 localization to the lysosomal membrane), or phospho‐cofilin (a Rho Kinase target) (Fig. 2A and B) was observed. Interestingly, levels of phospho‐MAPK were decreased more than twofold in the FLCN‐deficient cells; to our knowledge FLCN‐dependent changes in the MAPK pathway have not been previously reported. Cytochrome C oxidase subunit 4 (COX4), a mitochondrial protein which was previously found to be strikingly increased in FLCN‐deficient kidney and skeletal muscle cells (Hasumi et al. 2012), was not increased in FLCN‐deficient HBE cells.

SAEC with FLCN downregulation demonstrated distinct signaling changes when compared with HBE cells, with no change in phospho‐ACC, a twofold increase in phospho‐S6, and no change in phospho‐MAPK (Fig. 2C and D). These findings highlight what appear to be striking cell‐type‐specific effects of FLCN deficiency. Similarly to the HBE cells, but in contrast to other cell types, there was no change in phospho‐cofilin, p62/sequestosome 1, or Cox4 in SAEC with FLCN downregulation, compared to SAEC with control shRNA.

### Downregulation of FLCN impacts the expression of distinct TGF‐beta target genes in HBE and SAEC cells

**Figure 3.**
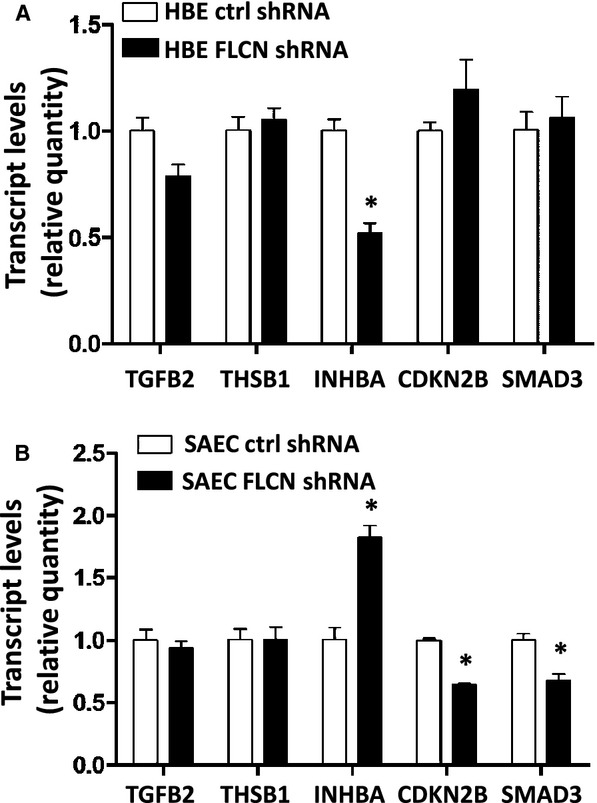
FLCN differentially regulates TGF‐beta target gene transcription in lung‐derived cells. mRNA from HBE (A) and SAEC (B) cells (three biologic replicates per shRNA cell line and three technical replicates) expressing FLCN shRNA or control shRNA was analyzed by qRT‐PCR for five TGF‐beta targets. *P < 0.05

FLCN‐deficient kidney‐derived cells and embryonic stem cells have low levels of TGF‐beta signaling components (INHBA, SMAD3) and low levels of TGF‐beta transcriptional targets (PAI‐1, Lefty) (Hong et al. 2010; Cash et al. 2011). We used quantitative RT‐PCR to measure the levels of three TGF‐beta pathway components (TGFB2, INHBA, and SMAD3) and two TGF‐beta targets (THBS1 and CDKN2B) which were previously found to be significantly lower in FLCN‐deficient kidney cancer cells and tumors (Hong et al. 2010). In FLCN‐deficient HBE cells, INHBA was decreased (Fig. 3A), while in FLCN‐deficient SAEC cells, INHBA was increased, and SMAD3 and CDKN2B were decreased (Fig. 3B), further highlighting the cell‐type‐ specific impact of FLCN deficiency.

### Downregulation of FLCN increases cell–cell adhesion in HBE cells

**Figure 4.**
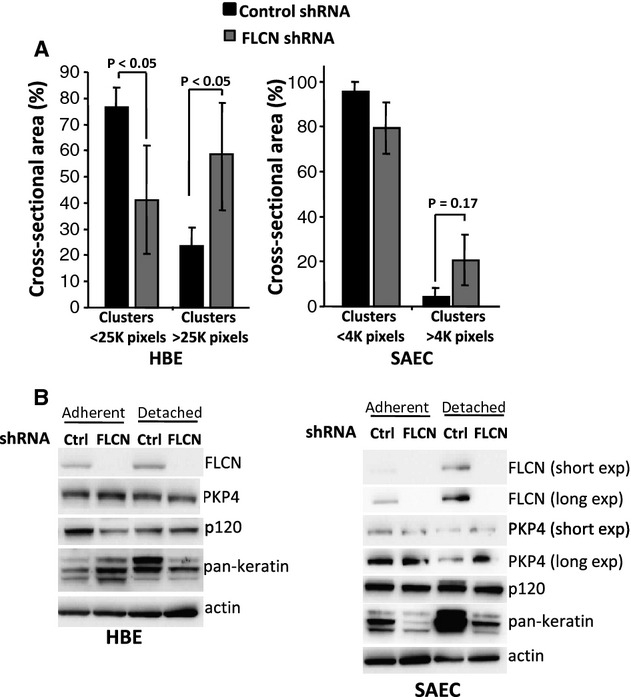
FLCN downregulation increases cell–cell adhesion in HBE cells. (A) HBE and SAEC cells with FLCN downregulation or control shRNA were grown as clusters in adherence‐free conditions for 96 h and then sheared using a standardized protocol (see Methods). (B) Immunoblot was used to compare levels of cell adhesion proteins in SAEC and HBE cells with control shRNA or FLCN shRNA cells grown in adherent monolayers or in detached conditions.

Previously, we found that loss of FLCN enhances cell–cell adhesion in kidney‐derived cells (UOK‐257 cells and HEK293 cells) grown in substrate‐free conditions (Medvetz et al. 2012). To determine whether FLCN deficiency enhances cell–cell adhesion in HBE and SAEC cells, we used a cellular aggregation assay in which cells were grown on an adherence‐free surface, and the size of cellular aggregates after shearing was measured using ImageJ (NIH, Bethesda, MD, USA) and Metamorph software (Molecular Devices, Sunnyvale, CA). Fifty‐eight percent of FLCN‐deficient HBE cells formed cellular clusters that were larger than 25,000 pixels; in contrast, only 23% of control shRNA cells formed clusters larger than 25,000 pixels (Fig. 4A; *P* < 0.05). These results indicate that FLCN loss increases cell–cell adhesion in HBE cells, consistent with our prior results in kidney‐derived cells. In SAEC, clusters tended to be 4–5 fold smaller than in HBE cells, and no difference in cluster size was detected between FLCN shRNA and control shRNA.

To determine whether loss of FLCN is associated with changes in proteins that regulate cell adhesion, we used western immunoblot to monitor levels of plakophilin 4 (PKP4), which directly interacts with FLCN (Medvetz et al. 2012; Nahorski et al. 2012), p120 catenin, which is the closest homolog of PKP4, and keratin. Protein levels were studied in both adherent and detached growth conditions. Interestingly, keratin levels were markedly diminished in FLCN‐deficient HBE and SAEC in detached growth conditions (Fig. 4B).

### Haploinsufficiency of *Bhd* does not cause airspace enlargement in mice

**Figure 5.**
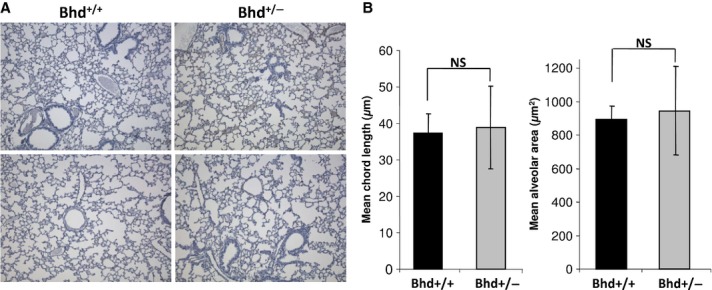
*Bhd* haploinsufficiency does not cause airspace enlargement at 5 months of age. (A) Representative Gills‐stained images of inflated lung sections from *Bhd*^+/+^ and *Bhd*^+/−^ mice. (B) Mean chord length and alveolar area in *Bhd*^+/−^ mice compared with littermate control *Bhd*^+/+^ mice (*n* = 5 per group). Data are presented as mean ± SD.

Human BHD patients (who have inactivation of one allele of the BHD gene) develop cystic airspace enlargement and are at an increased risk of pneumothorax. Mice with inactivation of one allele of the *Bhd* gene develop renal cysts and solid renal tumors with oncocytic features resembling chromophobe renal cell carcinoma (Luijten et al. 2013). To our knowledge, no prior studies have assessed whether *Bhd*^+/−^ mice spontaneously develop airspace enlargement. We inflated the lungs of *Bhd*^+/−^ mice and *Bhd*^+/+^ littermate controls in the AJ genetic background at 5 months of age, and measured alveolar chord length and alveolar area in a blinded manner on Gills‐stained inflated lung sections (Fig. 5A). No significant differences were observed in the mean alveolar chord lengths or the mean alveolar areas of *Bhd*^+/+^ and *Bhd*^+/−^ mice (Fig. 5B). Thus, loss of one *Bhd* allele does not result in the development of spontaneous airspace enlargement in mice at 5 months of age.

### Response to mechanical overdistension in *Bhd*^+/−^ mice

**Figure 6.**
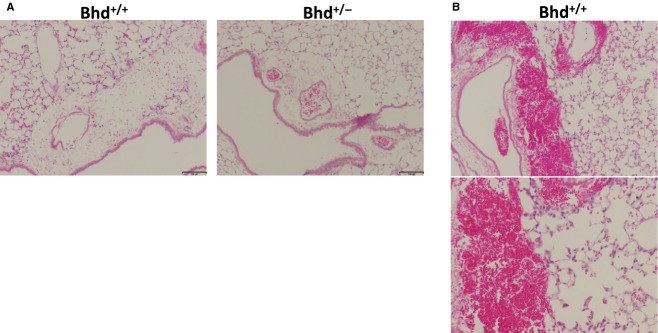
(A) Interstitial edema in *Bhd*^+/+^ and *Bhd*^+/−^ mice after 6 h of mechanical ventilation. (B) One mouse from the *Bhd*^+/+^ cohort had areas of hemorrhage after 6 h mechanical ventilation.

**Figure 7.**
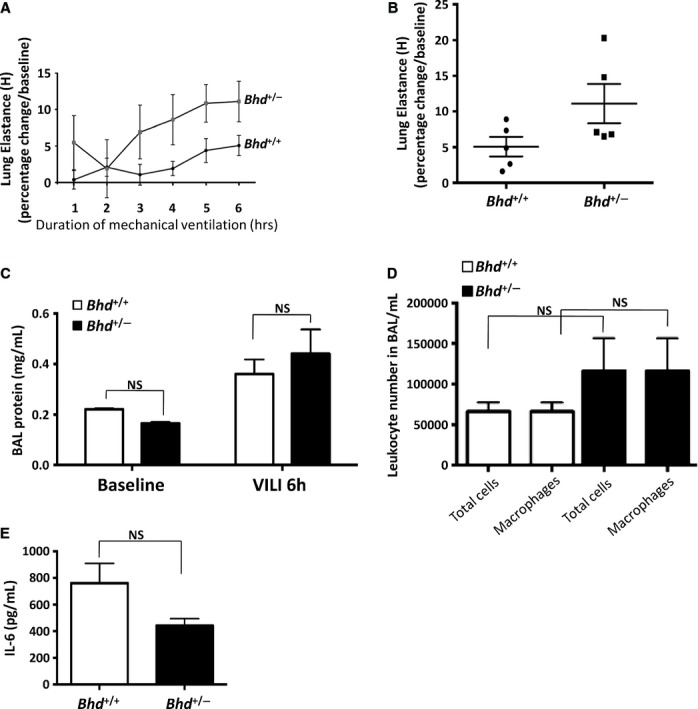
*Bhd*^+/−^ mice do not exhibit higher lung injury parameters following ventilation at high tidal volume. *Bhd*^+/−^ and *Bhd*^+/+^ littermate controls (*n* = 5 per group) were subjected to ventilation at high tidal volume (24 cc/kg) for 6 h. *Bhd*^+/−^ mice exhibited a trend toward increased lung elastance after ventilation (*P* = 0.08 at 6 h) compared with *Bhd*^+/+^ mice (A). Scatter plot of elastance measurements after 6 h of mechanical ventilation in *Bhd*^+/−^ mice compared with *Bhd*^+/+^ mice (B). BAL protein levels (C), BAL total leukocyte and macrophage numbers (D) and BAL IL‐6 (E) were measured following 6 h of mechanical ventilation. Data are presented as mean ± SD.

**Figure 8.**
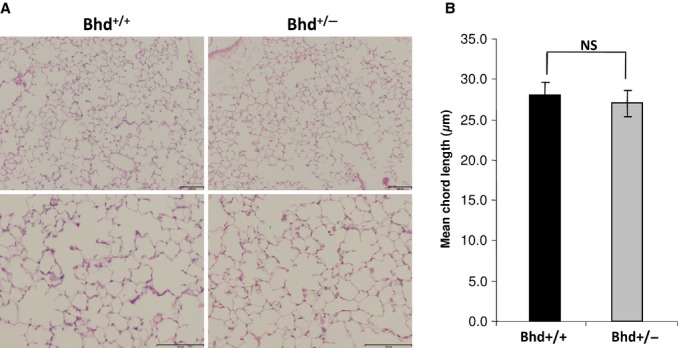
Mechanical ventilation in *Bhd*^*+/−*^ does not cause airspace enlargement. (A) Representative H&E‐stained images of inflated lung sections from *Bhd*^+/+^ and *Bhd*^+/−^ mice after 6 h of ventilation (lower and higher magnifications of representative fields are shown, scale bars are 100 *µ*m). (B) Mean chord length in *Bhd*^+/−^ mice compared with littermate control *Bhd*^+/+^ mice after 6 h of ventilation (*n* = 5 per group). Data are presented as mean ± SD.

Because of the enhanced cell–cell adhesion we observed in FLCN‐deficient HBE cells (Fig. 4), we hypothesized that the lungs of *Bhd*^+/−^ mice might be more susceptible to mechanical stretch‐induced forces (barotrauma). To test this hypothesis, *Bhd*^+/−^ and littermate control *Bhd*^+/+^ mice were subjected to mechanical ventilation at supraphysiologic tidal volumes (24 cc/kg for 6 h) using a Flexivent device. Mechanical ventilation induced interstitial edema in all animals (Fig. 6A); one mouse from the *Bhd*^+/+^ cohort exhibited multiple areas of hemorrhage (Fig. 6B). *Bhd*^+/−^ mice had a trend toward a twofold increase in lung elastance compared with *Bhd*^+/+^ wild‐type mice (Fig. 7A and B). No significant changes in bronchoalveolar lavage (BAL) protein (Fig. 7C), macrophages in the BAL fluid (Fig. 7D), or BAL IL‐6 levels (Fig. 7E) were observed in the *Bhd*^+/−^ mice after mechanical ventilation. We detected no difference in airspace enlargement between the two genotypes after 6 h of mechanical ventilation at high tidal volume (Fig. 8A and B).

## Discussion

Birt–Hogg–Dube syndrome is, to our knowledge, the only hereditary cancer syndrome with clinically significant pulmonary manifestations. It is puzzling that BHD patients develop tumors in the kidney and skin, but have a cystic phenotype in the lung. To elucidate the functions of FLCN in lung‐derived cells, we examined FLCN‐dependent phenotypes that have been documented in other cellular lineages, including proliferation, cellular signaling in the mTORC1, AMPK, and TGF‐beta pathways, and cell–cell adhesion. We found that FLCN downregulation in HBE cells increased cell–cell adhesion, consistent with our prior work in kidney‐derived cells, suggesting that regulation of cell–cell adhesion is a fundamental property of FLCN that could contribute to the lung phenotypes of human BHD. Importantly, an effect on cell–cell adhesion was not detected in FLCN‐deficient SAEC cells, suggesting that the effects of FLCN deficiency within the human lung are likely cell‐type‐specific and consistent with recent work in the mouse lung (Goncharova et al. 2014).

Because of the FLCN‐dependent alterations in cell–cell adhesion forces, we hypothesized that alterations in lung physiology would be observed when the lung epithelium of *Bhd*^+/−^ mice is exposed to stretch‐induced forces. Interestingly, the larger cysts in BHD patients tend to be in the lower lobes of the lungs (Agarwal et al. 2011; Tobino et al. 2011; Johannesma et al. 2014) where mechanical stress is the highest, consistent with a relationship between cyst pathogenesis and mechanical stress. To test the hypothesis that the lungs of Bhd^+/−^ mice are more vulnerable to stretch‐induced forces, we exposed *Bhd*^+/−^ mice to ventilation at super‐physiologic tidal volumes. No significant differences in lung physiology were observed, although there was a trend (*P* = 0.08) toward an increase in lung elastance after 6 h of mechanical ventilation. The trend toward increased elastance may reflect an underlying vulnerability of the *Bhd*^+/−^ lung epithelium to injury resulting from respiration‐induced physical forces because of alterations in cell–cell adhesion, but this will require further investigation. Because all cells in *Bhd*^+/−^ mice have inactivation of one allele of the *Bhd* gene, as do BHD patients, the impact of FLCN deficiency in this model could involve not only cell–cell adhesion and cellular signaling between the haploinsufficient cells of the lung epithelium but also lung capillary endothelial cells.

Several FLCN‐binding partners have been identified which could participate in the pathogenesis of pulmonary cysts in BHD, including FNIP1 and PKP4 (p0071). *Fnip1*^−/−^ mice develop normally and do not develop renal tumors (Baba et al. 2012), while *Bhd*^−/−^ embryos die at embryonic day 5.5 (Hasumi et al. 2009; Cash et al. 2011) and *Bhd*^+/−^ mice develop oncocytic renal tumors that resemble those in BHD patients (Hartman et al. 2009). The FLCN–PKP4 interaction (Medvetz et al. 2012; Nahorski et al. 2012) may mediate FLCN‐dependent cell–cell adhesion in HBE cells, since loss of PKP4 phenocopies loss of FLCN in other cell lineages (Medvetz et al. 2012). PKP4 directly interacts with VE‐cadherin, which is critical to the remodeling and integrity of endothelial cell–cell junctions, providing a potential between endothelial cell remodeling and the angiogenesis observed in human BHD cysts (Nishii et al. 2013). Both PKP4 and its closest homolog p120 catenin localize to adherens junctions and bind E‐cadherin. While no differences were observed in the protein level of PKP4 in FLCN‐deficient lung‐derived cells, the function of PKP4 may be impaired in cells lacking FLCN. Consistent with this notion, FLCN‐deficient mouse alveolar epithelial cells were recently found to have low levels of E‐cadherin (Goncharova et al. 2014).

No changes were observed in the median chord length in *Bhd*^+/−^ mice compared to *Bhd*^+/+^ controls at 5 months of age. Renal tumorigenesis in human BHD requires inactivation of both alleles of the BHD gene (Vocke et al. 2005). It is possible that a “second hit” genetic event resulting in loss of function of both copies of FLCN is required for lung cystogenesis. The primary phenotype of loss of FLCN in mice is renal cystogenesis (Baba et al. 2008; Chen et al. 2008; Hartman et al. 2009). The relationship between the mechanisms of cyst pathogenesis in the kidney versus the lung is unknown. Cyst initiation in both the kidney and lung may initiate during development due to altered epithelial integrity together with the fundamental role of FLCN in embryonic stem cell differentiation (Betschinger et al. 2013).

In conclusion, we report that FLCN‐deficient HBE cells exhibit aberrant cell–cell adhesion and that the effects of FLCN deficiency on the mTORC1 and AMPK pathways differ strikingly between HBE and SAEC cells, revealing cell‐type‐specific effects of FLCN deficiency. *Bhd*^+/−^ mice do not exhibit baseline airspace enlargement. *Bhd*^+/−^ mice exhibit a trend toward increased elastance in response to stretch‐induced forces applied through mechanical ventilation at high tidal volume. Further investigation, including the use of cells cultured on an air‐liquid interface and examination of cell–cell adhesion on lung cells derived from Bhd+/− mice, will be required to fully understand the physiologic consequences of FLCN deficiency and the relevance of mechanical force to the lung phenotypes in BHD patients.

## Conflict of Interest

None declared
